# Supplementary material for: Convergent and distributed effects of the 3q29 deletion on the human neural transcriptome
Source: Transl Psychiatry. 2021 Jun 15;11:357. doi: 10.1038/s41398-021-01435-2 (PMC8206125; doi:10.1038/s41398-021-01435-2)
Supplement: Supplementary file 2 — Supplemental Figures [file 41398_2021_1435_MOESM2_ESM.pdf]

Supplemental Figures

Convergent and distributed effects of the 3q29 deletion on the human neural transcriptome

Sefik & Purcell et al.

Table of contents

Fig. S1. Tissue sample attributes and donor phenotypes of the GTEx dataset used for reference network construction…..2
Fig. S2. Pre-processing of the reference dataset: outlier removal…..3
Fig. S3. Determination of the soft-thresholding power (β) in WGCNA……………………..…..…..4
Fig. S4. Determination of network reproducibility and module preservation in an independent test dataset…………………….……………………………………………..……………………....5
Fig. S5. Individual module-preservation and quality statistics underlying composite Zsummary scores………………………………………………………………………………………… 6-7
Fig. S6. Publication numbers for 3q29 genes and historic schizophrenia spectrum disorder candidate genes……………………………………………………………………………..……..…8
Fig. S7. Overlap between known protein-protein interactions (PPI) and gene co-expression patterns of 3q29 interval genes………………………………………………………………..….…9
Fig. S8. STRING protein-protein interaction (PPI) networks of genes co-expressed in 3q29 modules. ……………………………………………………………………………..……10-16
Fig. S9. Amalgamated illustration of top biological processes / pathways enriched in 3q29 modules.……………………………………………………………………………………….…17

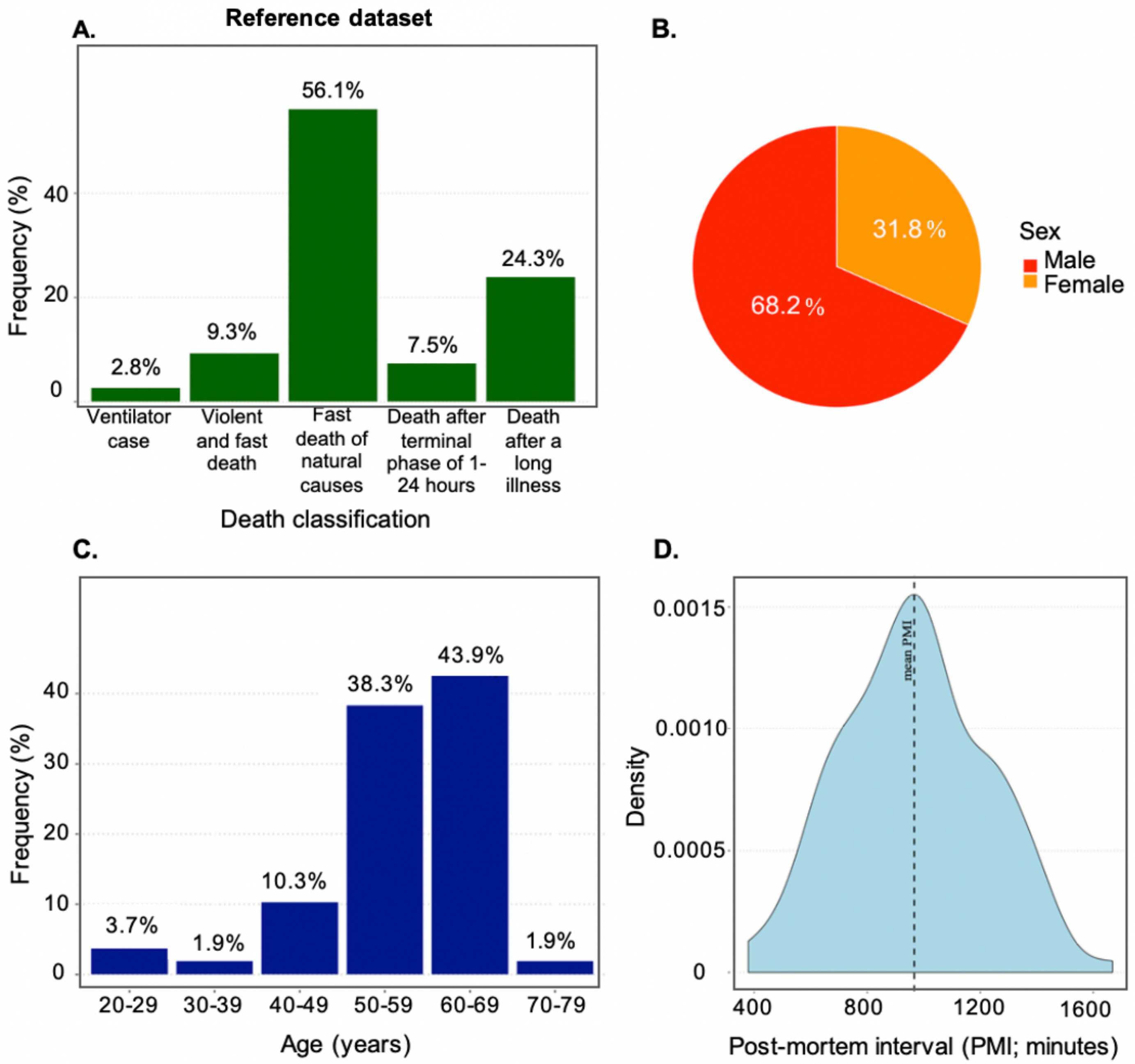

Fig. S1. Tissue sample attributes and donor phenotypes of the GTEx dataset used for reference network construction (*N* = 107). (A) Frequency distribution of donors' death-classification based on the 4-point Hardy scale. Majority of tissue samples (56.1%) were obtained from donors whose deaths were classified as "fast death of natural causes", which encompasses sudden (unexpected) deaths of people who had been reasonably healthy, following a terminal phase of <1 hour (e.g., sudden death from myocardial infarction). **(B)** Frequency distribution of donors' sex. Male:female ratio = 2.1:1.0. **(C)** Frequency distribution of donors' age-group based on 4-year intervals determined by the GTEx project. Majority of donors (82.2%) were in the age-range of 50-69 years at the time of death. **(D)** Sample density plot of post-mortem interval (PMI), indicating time elapsed between donor's death and final tissue stabilization. Mean sample PMI (sd) = 966.2 (254.3) minutes.

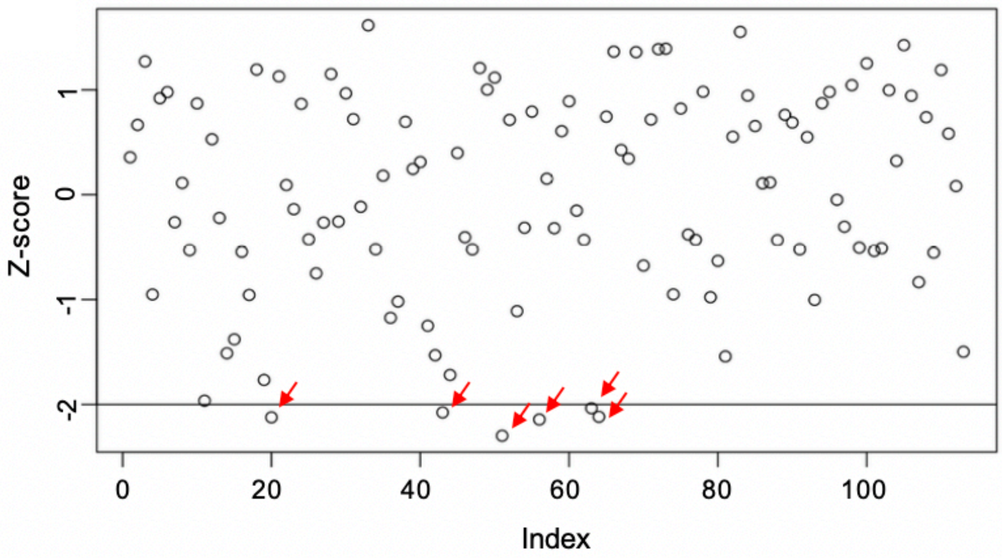

Fig. S2. Pre-processing of the reference dataset: outlier removal. 6 outlier samples (red arrows) were removed from the reference dataset (GTEx Project) to prevent an outlier-driven bias in gene co-expression network construction. Inter-sample correlation (ISC) was used as the statistical diagnostic for identifying samples with divergent gene expression profiles. ISC was defined as the Pearson's correlation between pairs of samples across the expression levels of all detected genes. Samples with a mean ISC greater than 2 standard deviations away (black line) from the mean of the sample-set were removed, bringing the sample size used for weighted gene co-expression analysis to 107.

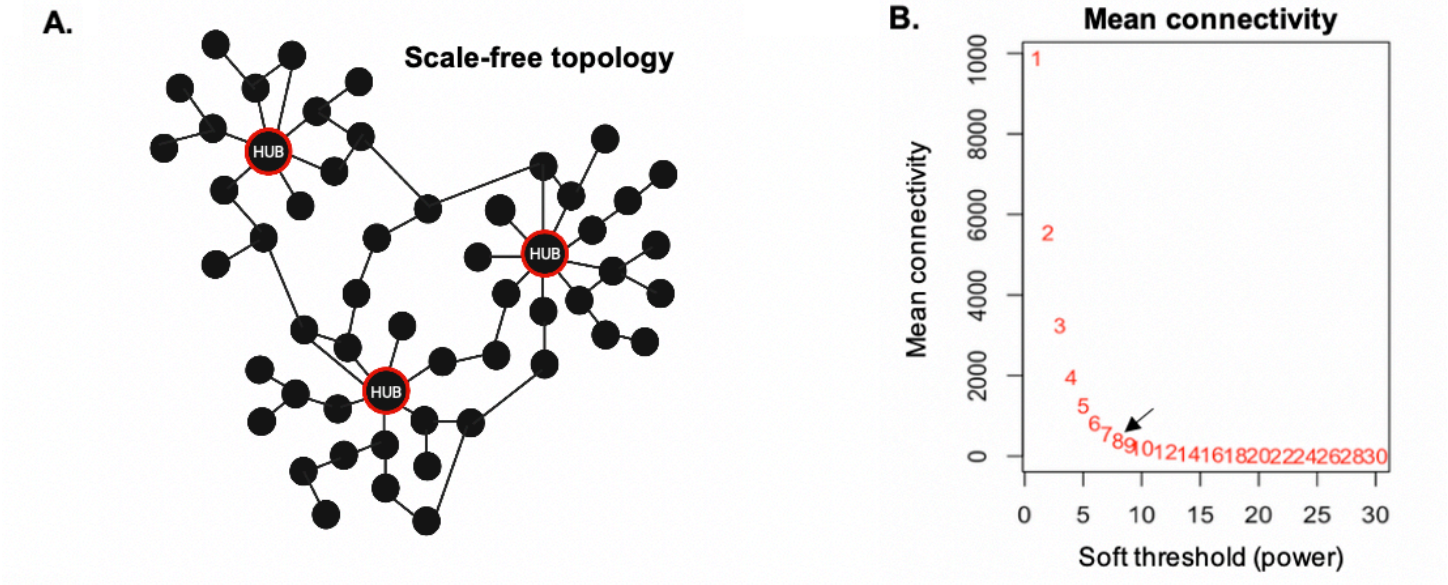

C.

Power	SFT R^2	Slope	Truncated R^2	Mean k	Median k	Max k
1	0.6	18.9	0.8	9890	9880	11100
2	0.5	7.0	0.9	5520	5460	7100
3	0.3	3.2	0.9	3200	3100	4740
4	0.0	0.5	0.8	1930	1810	3370
5	0.2	-1.0	0.8	1200	1090	2520
6	0.6	-1.6	0.9	773	674	1940
7	0.7	-1.8	1.0	513	430	1540
8	0.8	-1.8	1.0	351	281	1250
9	0.8	-1.8	1.0	247	186	1020
10	0.9	-1.8	1.0	178	125	854
12	0.9	-1.7	1.0	98	60	613
14	0.9	-1.8	1.0	58	30	459
16	0.9	-1.8	1.0	36	16	353
18	0.9	-1.7	1.0	24	9	278
20	0.9	-1.7	1.0	16	5	222
22	0.9	-1.7	1.0	11	3	180
24	0.9	-1.7	1.0	8	2	148
26	0.9	-1.7	1.0	6	1	123
28	0.9	-1.7	1.0	4	1	103
30	0.9	-1.7	1.0	3	0	87

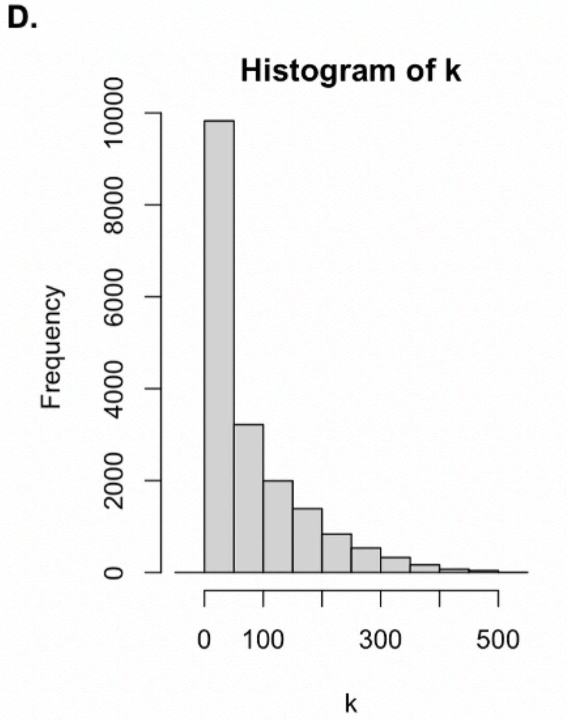

Fig. S3. Determination of the soft-thresholding power (β) in WGCNA. (A) Schematic of a representative scale-free network, whose degree distribution follows a power-law**:** most nodes have one or two connections but a few highly connected nodes (hub genes; marked by a red frame) have a large number of connections. Scale free topology (SFT) is a unifying property of biological networks in nature (Barabasi et al., 2004*). **(B)** Mean connectivity (k) as a function of different β values. Mean k decreases as β increases. The arrow marks the β value used in this study. **(C)** List of SFT fitting indices for a wide range of β values. The highlighted row indicates the β used in this study. Given the necessary trade-off between SFT index R^2 and mean connectivity (k), a β of 8 was identified as the lowest possible power yielding a degree distribution that results in approximate SFT (R^2 fit index = 0.8), while maintaining relatively high mean connectivity (mean k > 100), enabling the detection of modules and hub nodes. **(D)** Histogram of connectivity (k) distribution when a β of 8 was chosen for defining the adjacency matrix. The frequency distribution of k shows a large number of lowly connected genes and a small number of highly connected genes, indicating that the resulting network follows the SFT criterion.

* Barabasi AL, Oltvai ZN. Network biology: understanding the cell's functional organization. *Nat Rev Genet* 2004; **5**(2)**:** 101-113.

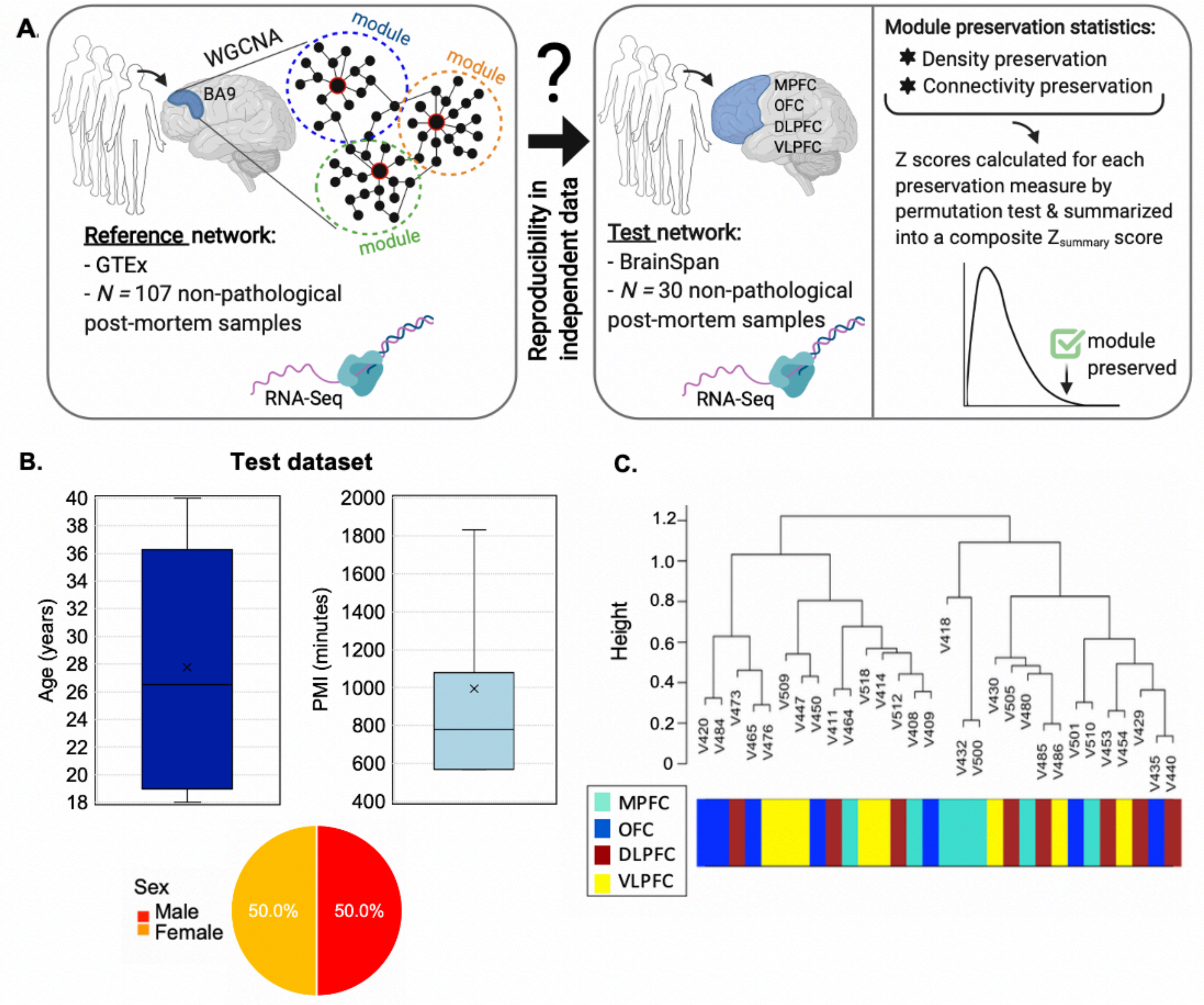

Fig. S4. Determination of network reproducibility and module preservation in an independent test dataset. (A) Illustration of the network reproducibility approach used in this study. Preservation of all network modules that were identified in the reference dataset (GTEx Project, N=107) was established in an independent, demographically comparable test dataset obtained from the BrainSpan Project (N=30). Both transcriptomic datasets were corrected for covariance mediated by sex, age and post-mortem interval (PMI). **(B)** Tissue sample attributes and donor phenotypes of the BrainSpan test dataset. The dark blue box-plot indicates the distribution of donors' age at death (in years). Median age = 27 years (range: 18-40 years). Mean age (sd) = 28 (8.4) years and corresponds to the cross mark. The light blue box-plot indicates the distribution of PMI in the test dataset. Median PMI = 780 minutes. Mean PMI (sd) = 994 (446.2) minutes. Pie chart indicates the percentage breakdown of donors' sex. Male:female ratio = 1:1. **(C)** Sample-level dendrogram of the test dataset plotted by hierarchical clustering of 30 non-pathological post-mortem samples obtained from four subregions of the prefrontal cortex (PFC) from male and female adults with no known history of neurological or psychiatric disorder. Clustering was conducted on normalized and residualized gene expression values for 18,339 protein-coding genes. Color bar below the dendrogram indicates tissue type corresponding to four subregions of the PFC that were pooled to derive the test dataset. The resulting dendrogram reveals no distribution bias associated with tissue-subtype in sample-level clustering patterns. OFC: orbital frontal cortex, DLPFC: dorsolateral PFC, VLPFC: ventrolateral PFC, MPFC: medial PFC.

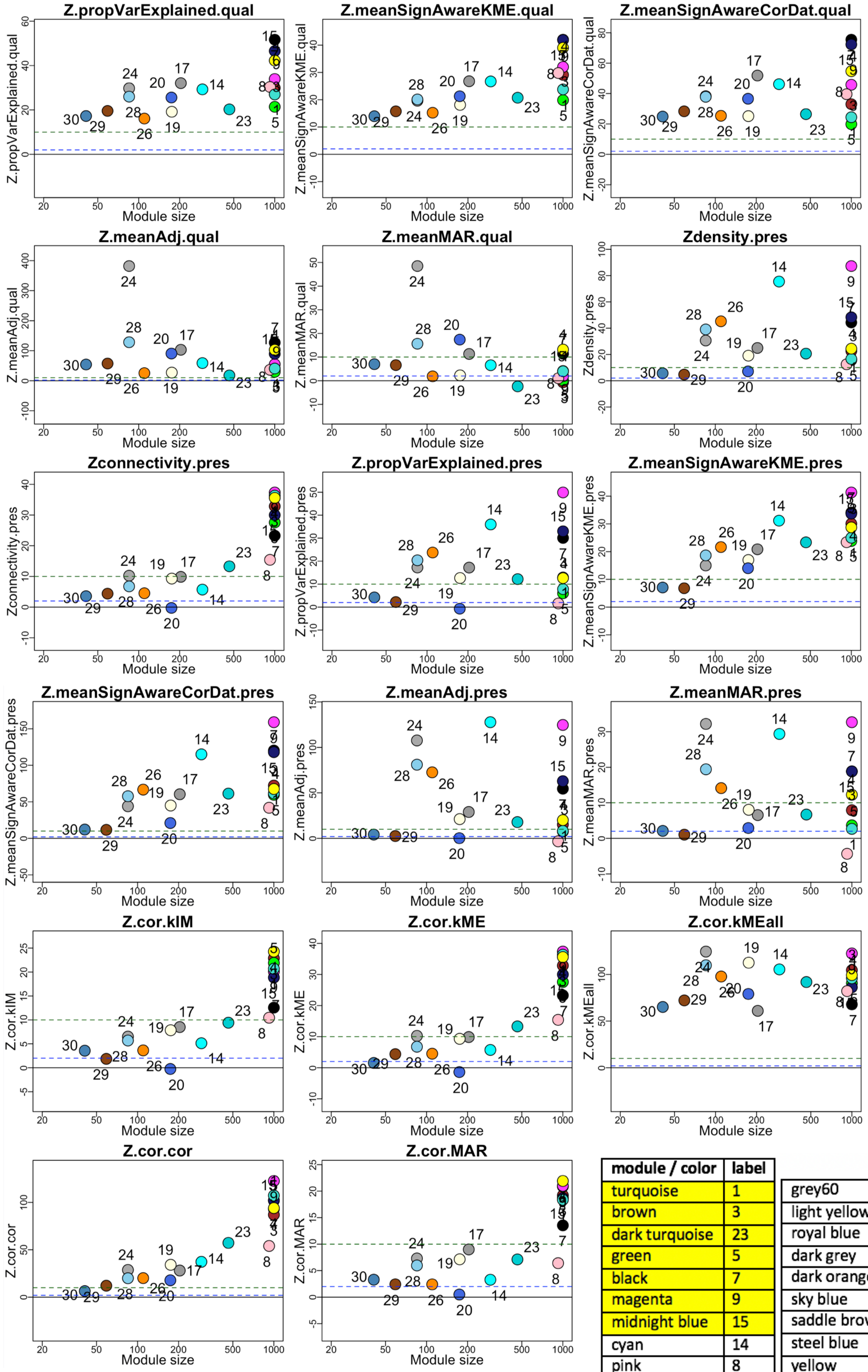

module / color	label
turquoise	1
brown	3
dark turquoise	23
green	5
black	7
magenta	9
midnight blue	15
cyan	14
pink	8

grey60	17
light yellow	19
royal blue	20
dark grey	24
dark orange	26
sky blue	28
saddle brown	29
steel blue	30
yellow	4

Fig. S5. Individual module-preservation and module-quality statistics underlying composite Zsummary scores. Multiple density-based and connectivity-based module-preservation statistics were assessed to determine the distinct properties of network structure preserved between the reference and test networks. In line with the composite $Z_{summary}$ statistic, individual module-preservation statistics mostly converged on the finding that 1) nodes sharing the same module in the reference network remain highly connected in the test network and 2) connectivity patterns between nodes underlying the reference network remain similar in the test network. Similarly, multiple module-quality based statistics were assessed to determine how distinct individual modules were from all other modules in the reference network. In line with the composite $Z_{summary}$ statistic, the majority of the evaluated module-quality statistics indicate robust module definitions across networks created from random splits of the original reference data. Permutation tests were performed to adjust the observed preservation and quality statistics of each module for random chance by defining Z statistics. Zstatistic < 2 (blue dotted line): no evidence for preservation/quality; 2 < Zstatistic < 10 (green dotted line): moderate evidence for preservation/quality; Zstatistic > 10: strong evidence for preservation/quality. Refer to Langfelder et al. (2017)* for a detailed description of the individual module preservation and quality statistics plotted above. Both numeric and color-based labels were used to mark individual modules. The coding system corresponding to module labels is provided in the bottom right corner of the figure; labels highlighted in yellow indicate the 7 modules harboring 3q29 interval genes.

* Langfelder P, Luo R, Oldham MC, Horvath S. Is My Network Module Preserved and Reproducible? *Plos Computational Biology* 2011; **7**(1).

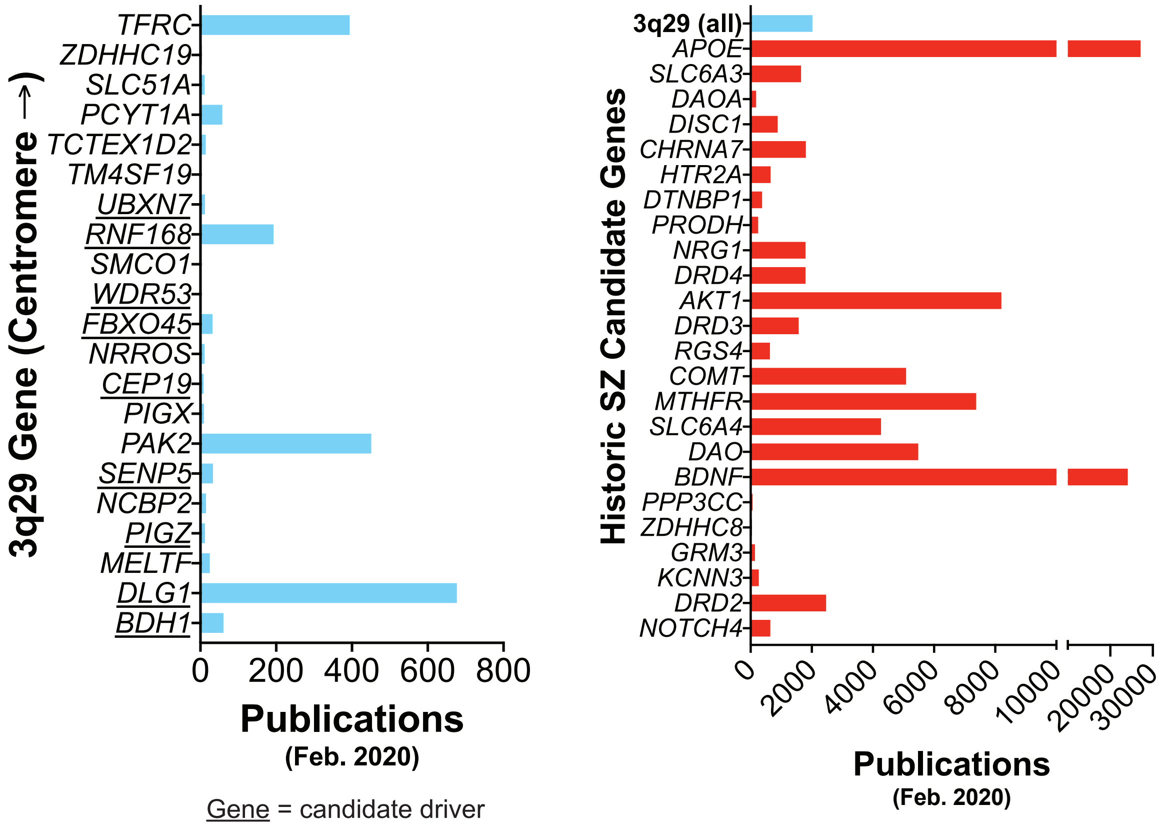

Fig. S6. Publication numbers for 3q29 genes and historic schizophrenia spectrum disorder candidate genes. The total number of PubMed articles retrieved with a search for the symbol of each gene is shown above. Notably, of the prioritized driver genes (underlined) identified in this study (left), only *DLG1* has a publication history that compares favorably with historic schizophrenia spectrum disorder (SZ) candidate genes (right) that were typically identified by genetic linkage and association studies. The majority of 3q29 interval genes remain understudied.

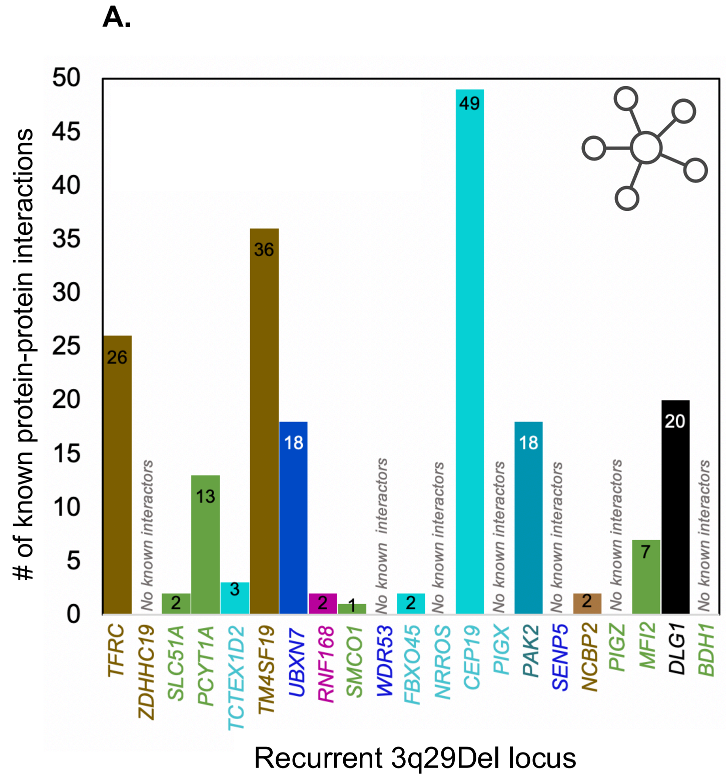

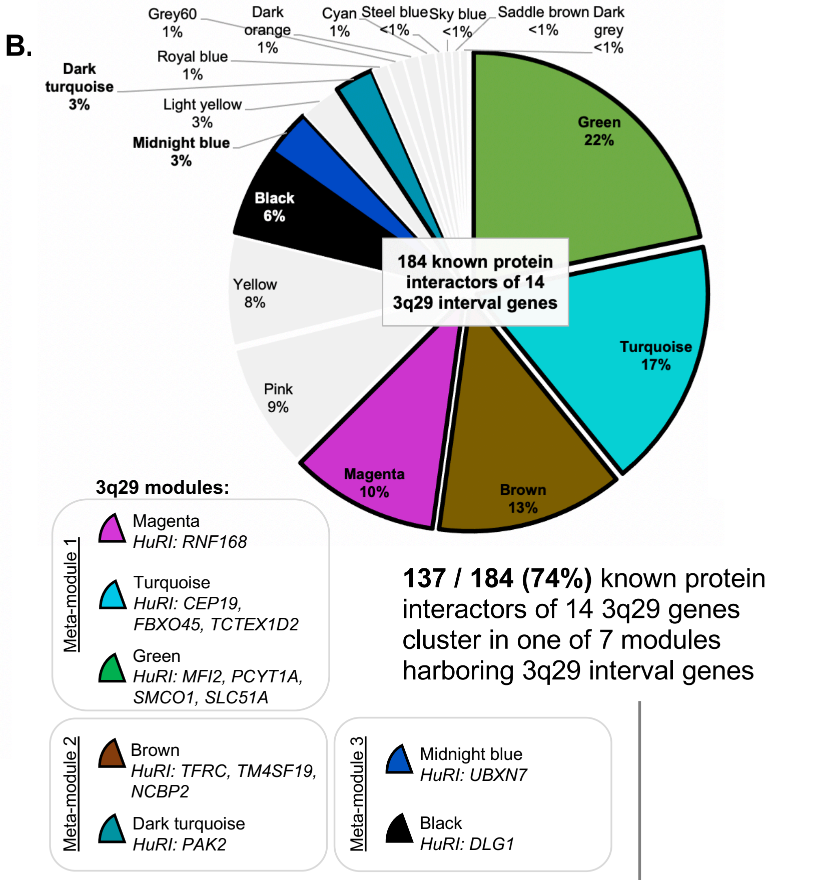

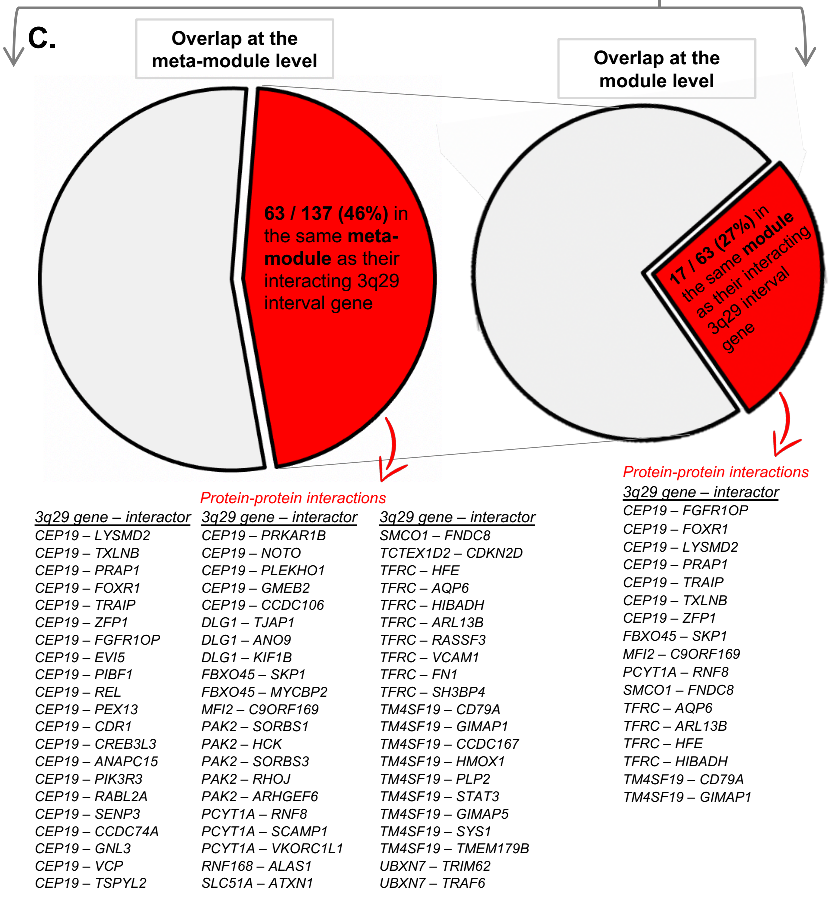

Fig. S7. Overlap between known protein-protein interactions (PPI) and gene co-expression patterns of 3q29 interval genes. (A) Number of known binary protein interactors of 3q29 interval genes curated from the Human Reference Protein Interactome (HuRI) database. Of the 21 protein coding genes located in the 3q29 interval, only 14 were found to have an entry on HuRI, 50% of which have less than 8 known proteome-wide interactors. X axis reflects the chromosomal order of the 3q29Del locus from centromere to telomere. Colors represent gene co-expression network modules. **(B)** Distribution of known protein interactors of 3q29 interval genes across our gene co-expression network. A total of 193 distinct protein interactors were identified on HuRI for 14 3q29 interval genes (after removing duplicates), 184 of which were identified as a node in our gene co-expression network. Of these 184, 137 (74%) were found to cluster in one of seven modules harboring 3q29 interval genes. **(C)** Overlap between 3q29 interval genes and their protein interactors at the module and meta-module levels of gene co-expression network organization. 46% of the protein interactors identified in 3q29 modules share the same meta-module as their interacting 3q29 interval gene, 27% of which further show an overlap at the module level.

Fig. S8. STRING protein-protein interaction (PPI) networks of genes co-expressed in 3q29 modules. PPI network of genes co-clustering in the **(A)** black, **(B)** midnight blue, **(C)** brown, **(D)** magenta, **(E)** dark turquoise, **(F)** green, and **(G)** turquoise modules are visualized by node-link diagrams. Network nodes represent protein products of genes that were found to co-cluster in the same gene co-expression network modules as the 21 protein-coding 3q29 interval genes. Edges represent PPI curated from the STRING database (https://string-db.org/). Line thickness is proportional to the strength of available evidence for each PPI. For clear and effective visualization, only nodes from seven distinct 3q29 modules that are part of a known physical protein complex and are connected by at least a "high confidence" (0.7) strength of evidence for interaction are included in these illustrations. Based on these criteria, disconnected nodes were discarded from the graphs.

A. Black module

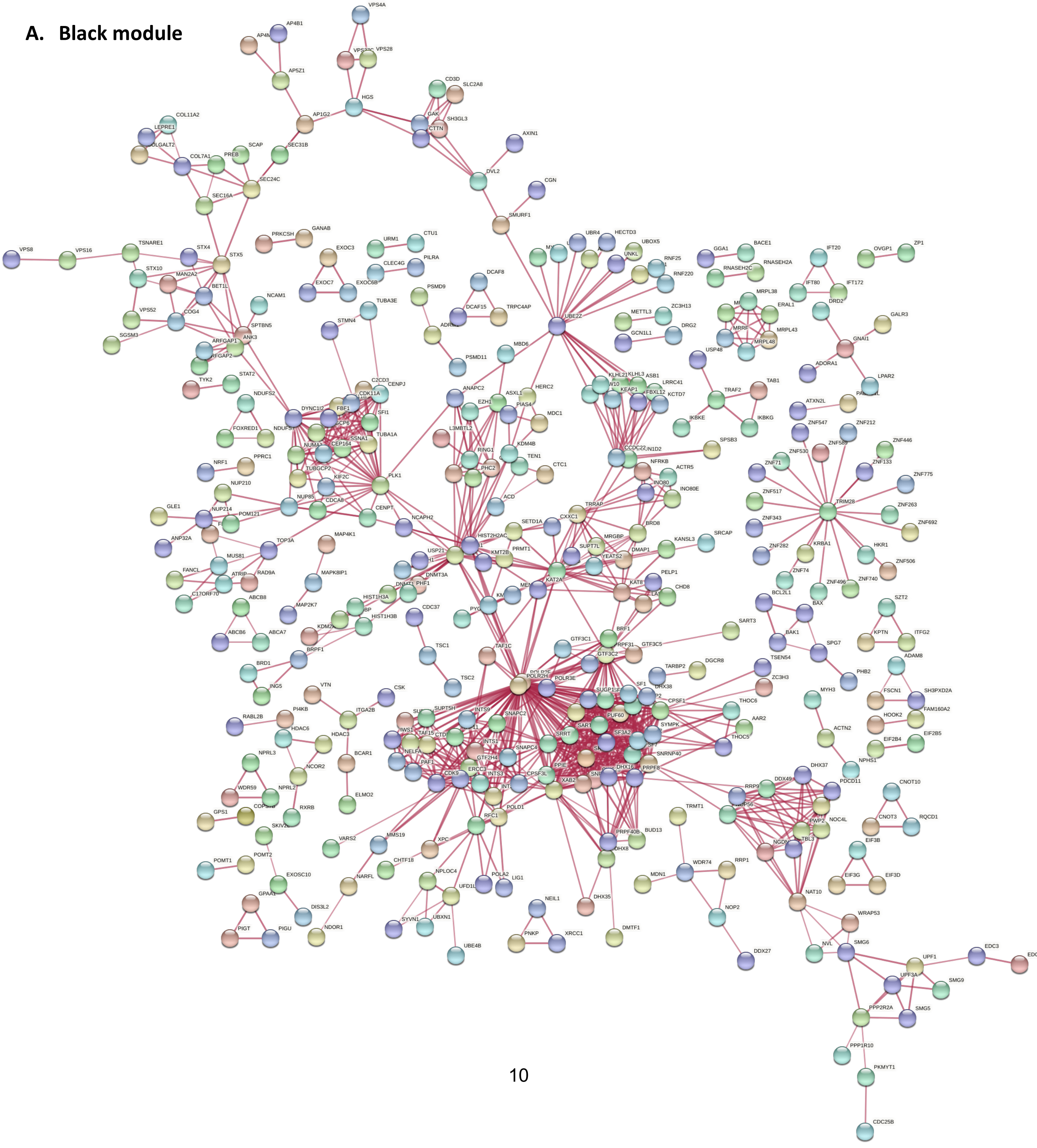

B. Midnight blue module

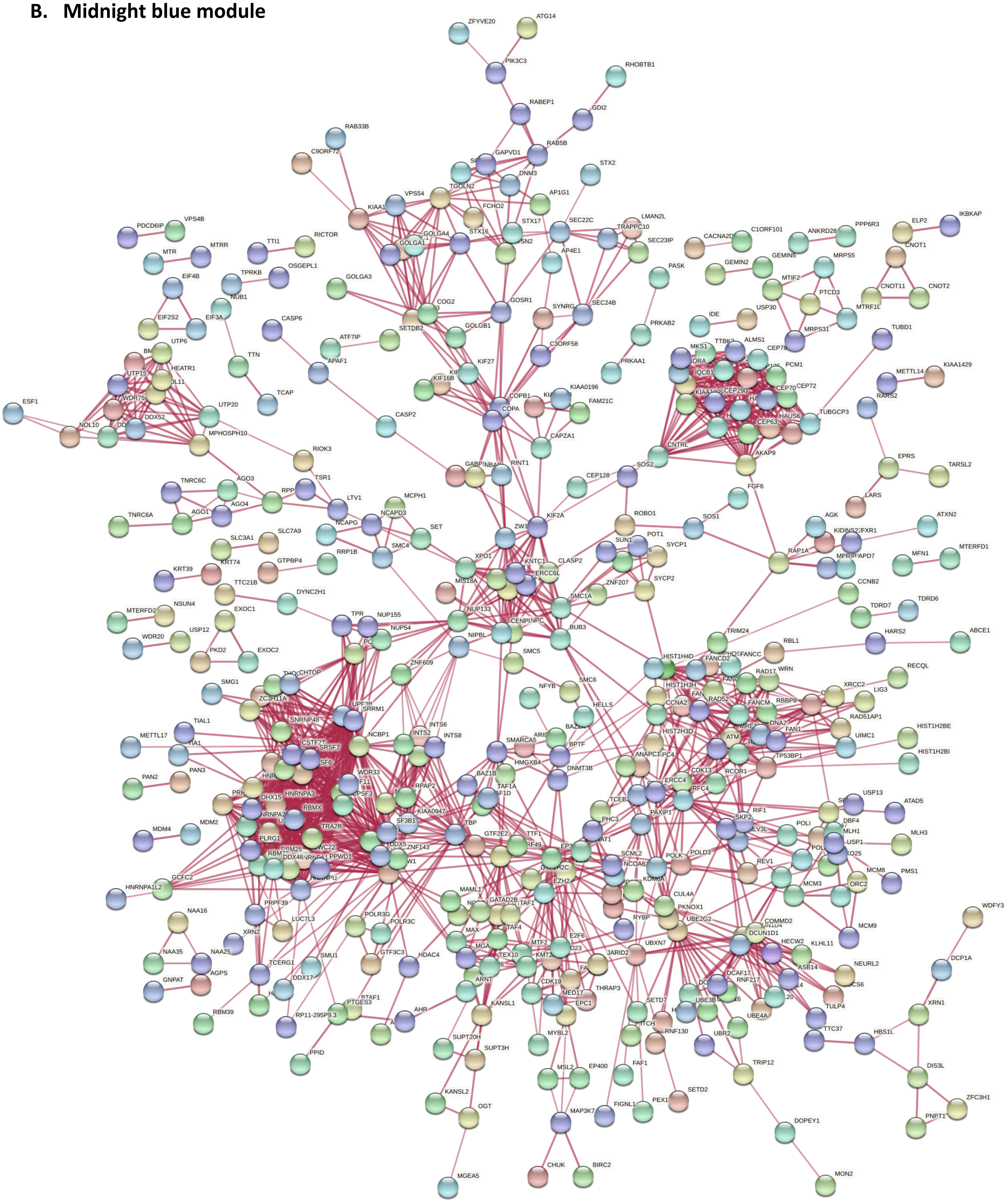

C. Brown module

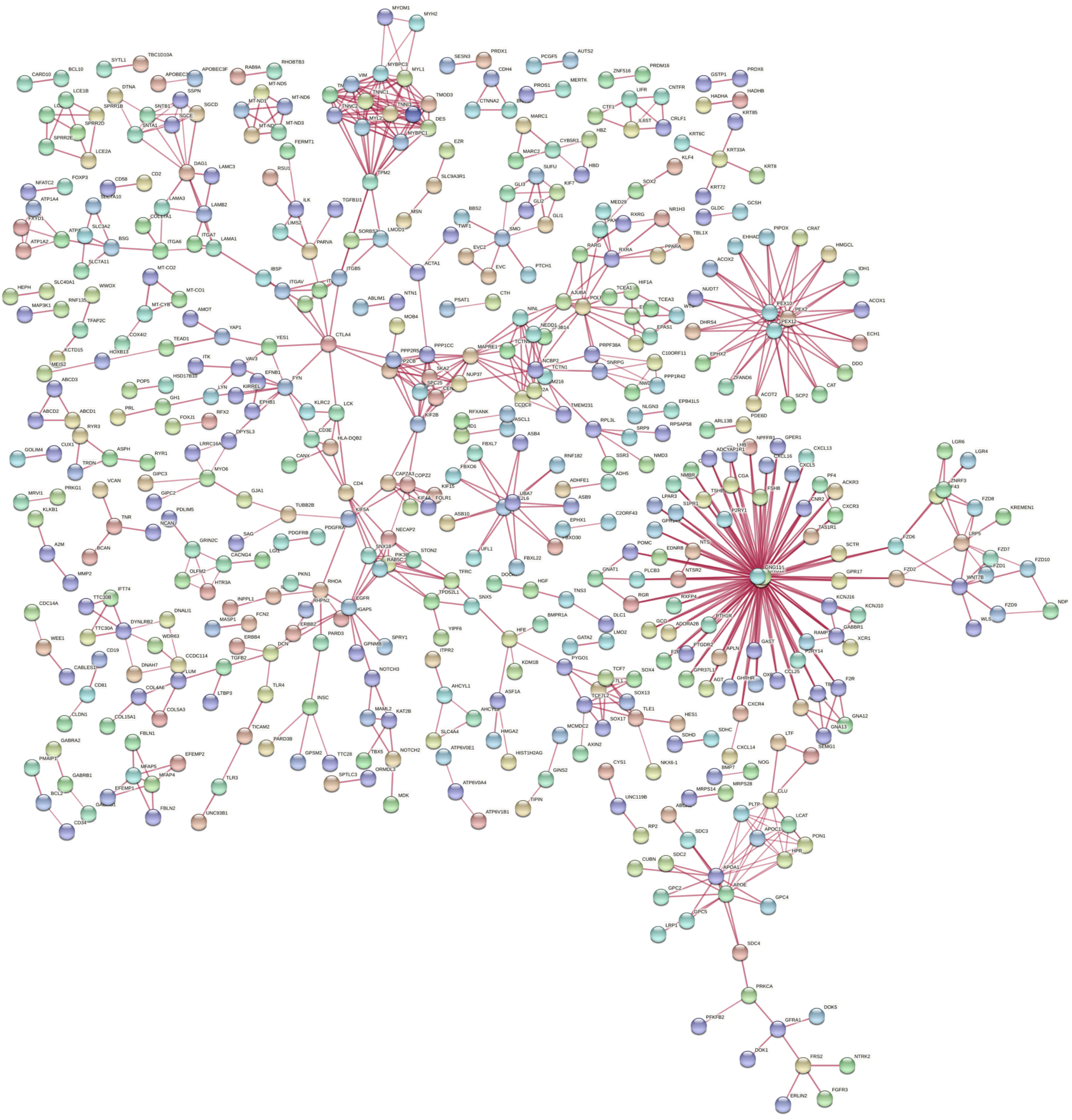

D. Magenta module

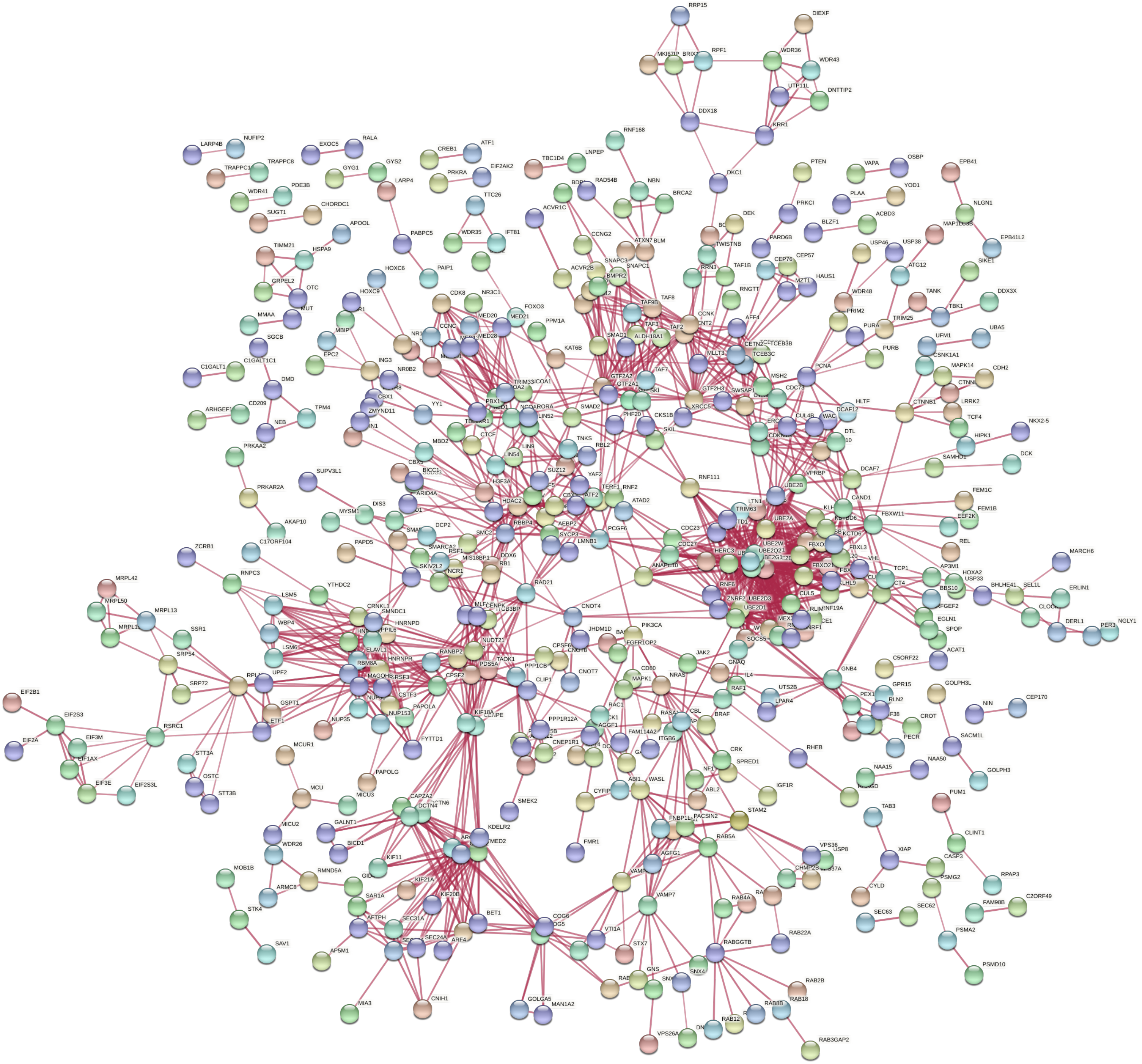

E. Dark turquoise module

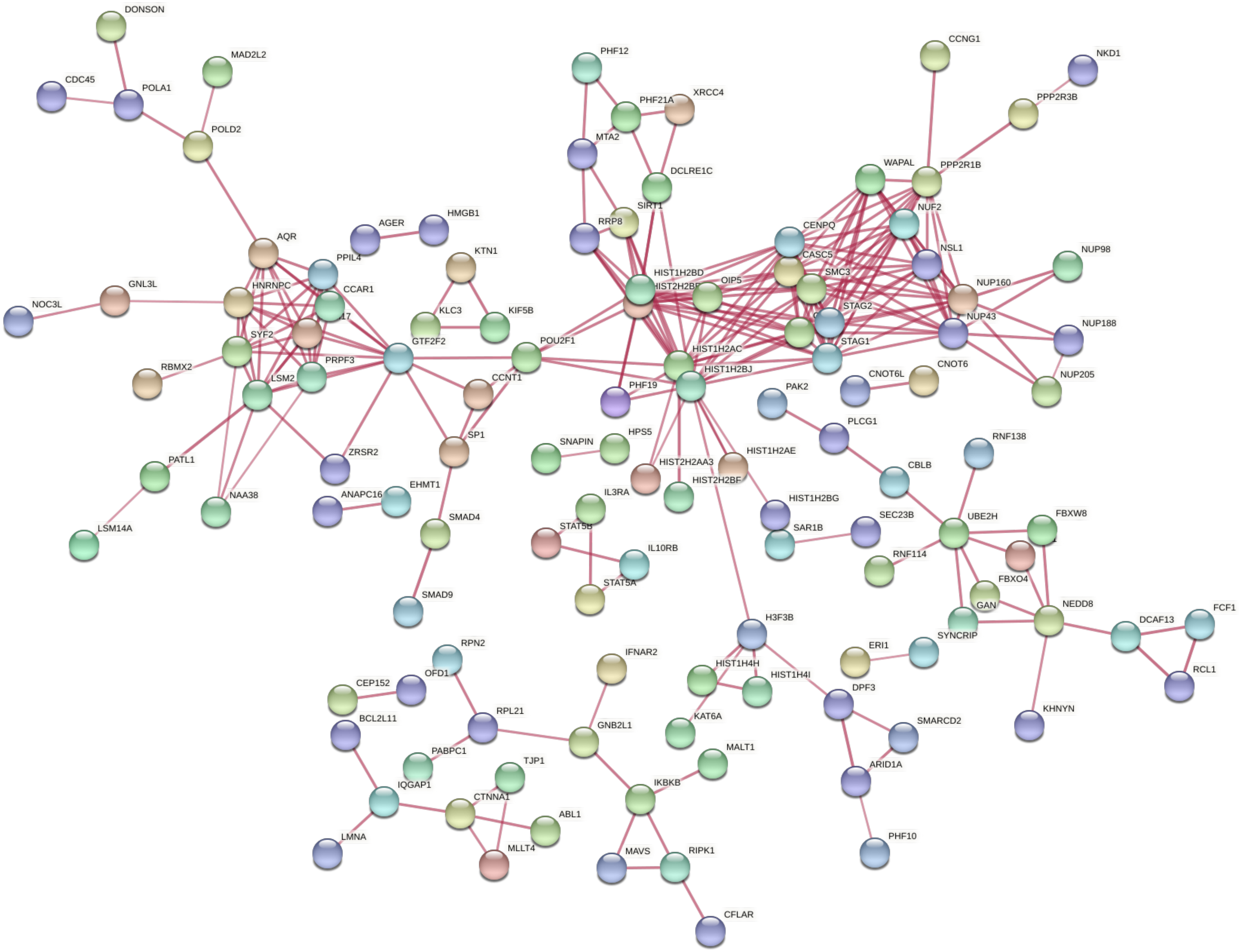

F. Green module

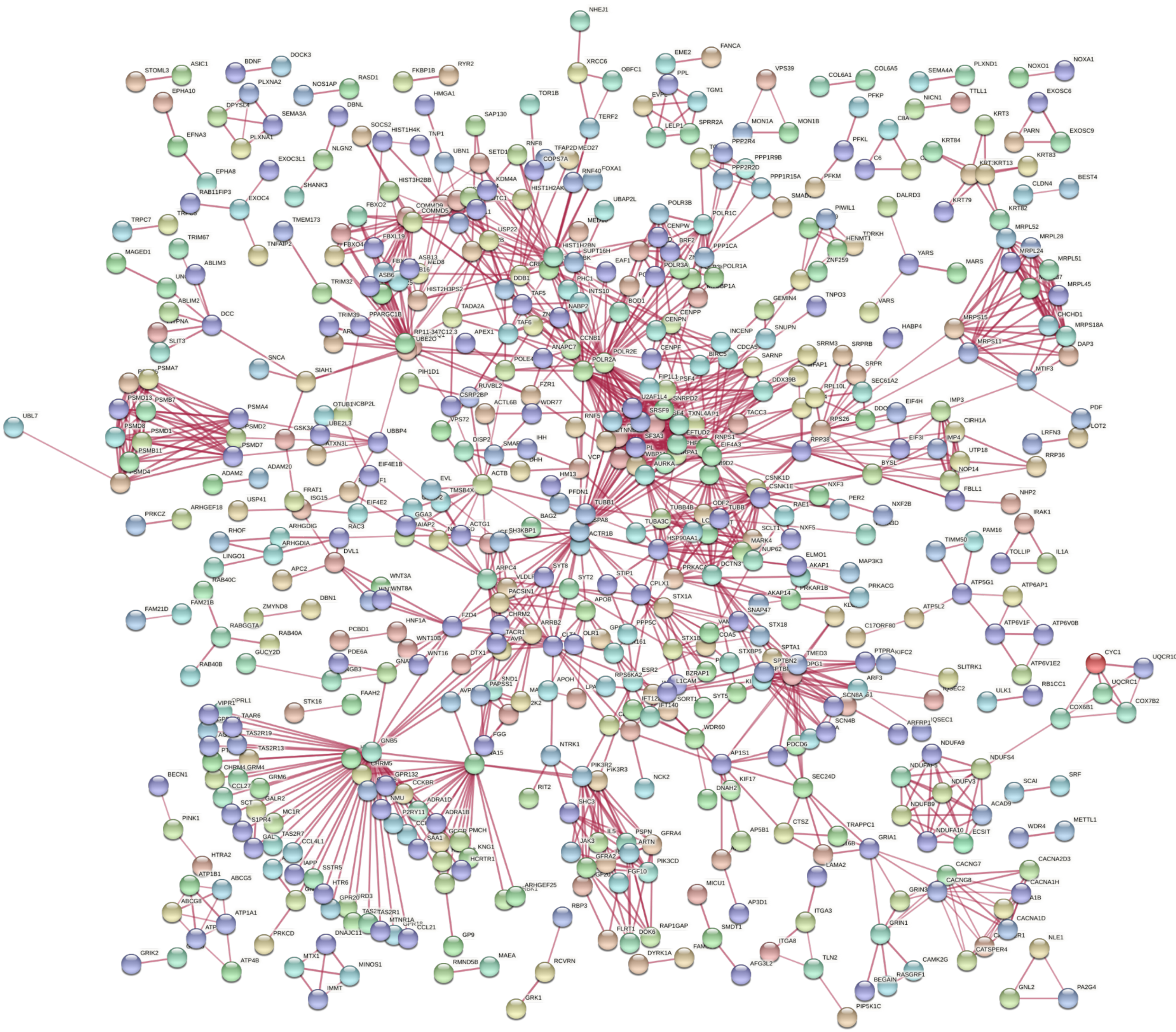

G. Turquoise module

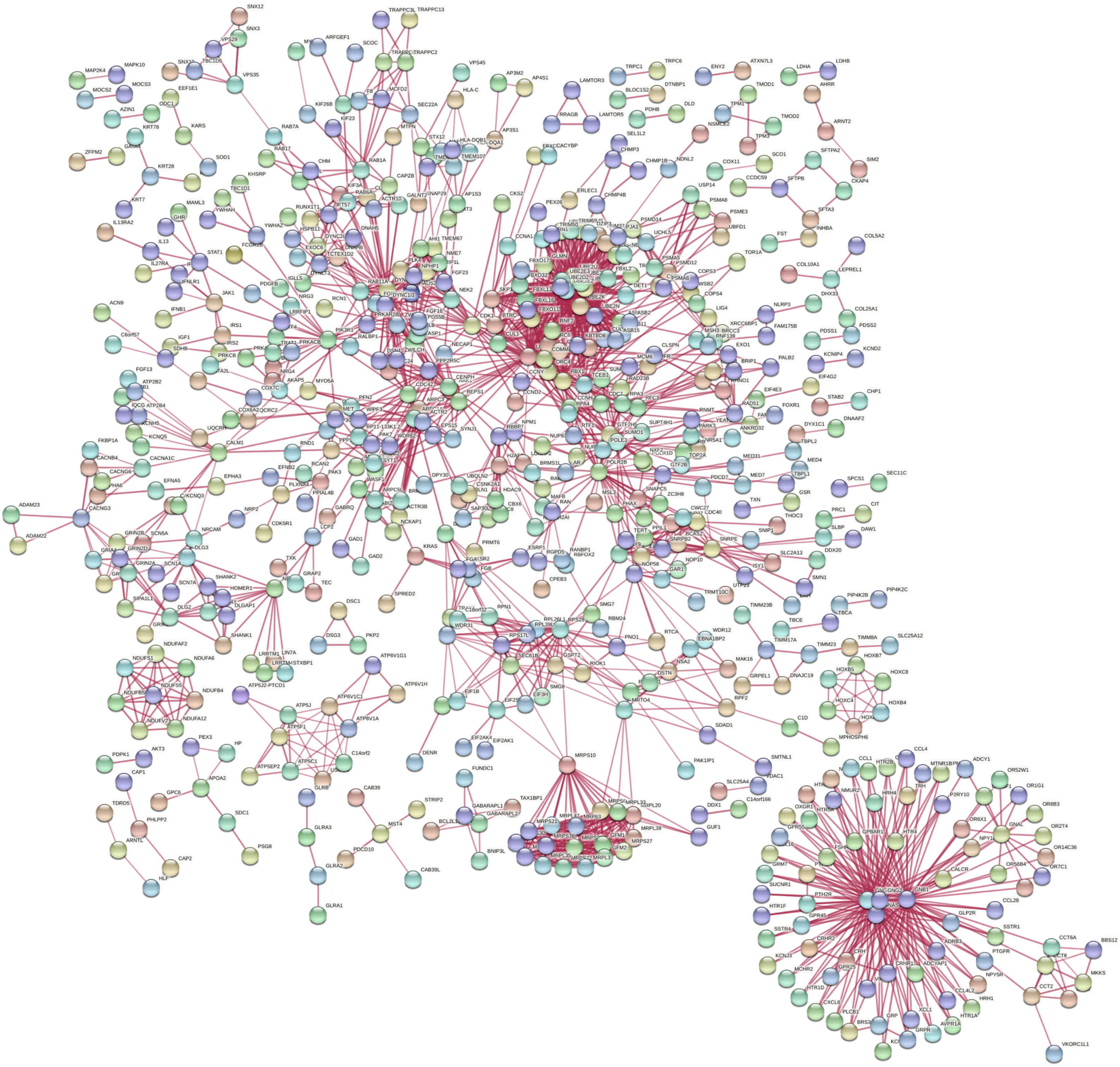

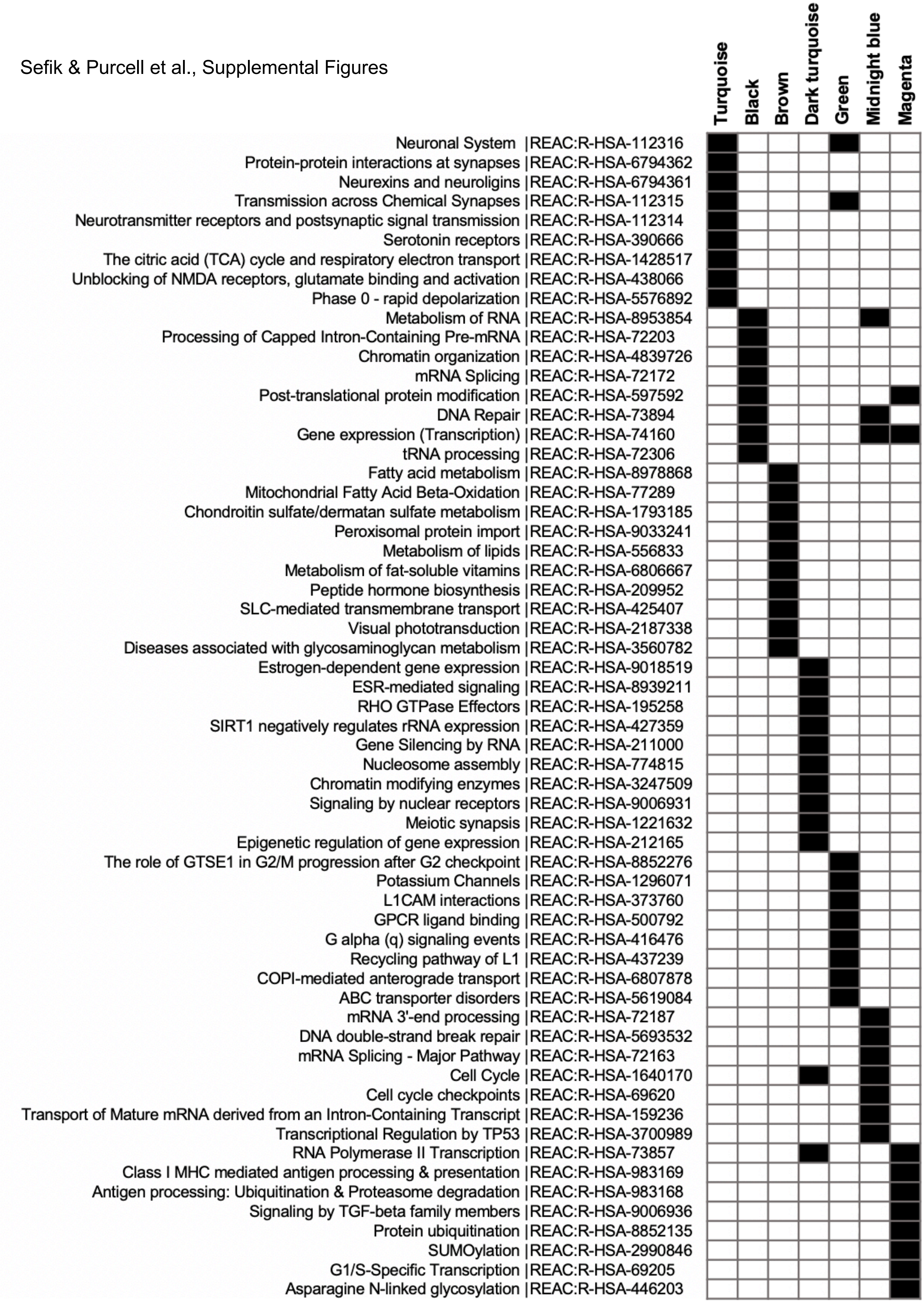

Fig. S9. Amalgamated illustration of top biological processes / pathways enriched in 3q29 modules. Enrichment plots from Fig. 2d have been combined in this discrete heatmap to illustrate the distinct vs. shared enrichment profiles of the top Reactome terms overrepresented in different 3q29 modules. Black cells indicate significant enrichment at adjusted-$P < 0.05$.
